# A quantitative risk assessment approach for mosquito-borne diseases: malaria re-emergence in southern France

**DOI:** 10.1186/1475-2875-7-147

**Published:** 2008-08-01

**Authors:** Nicolas Ponçon, Annelise Tran, Céline Toty, Adrian JF Luty, Didier Fontenille

**Affiliations:** 1 Institut de recherche pour le développement, UR016, 911 avenue Agropolis, BP64501, 34394, Montpellier, cedex 5, France; 2 Centre de coopération internationale de la recherche agronomique pour le développement, UPR22, campus de Baillarguet, 34398, Montpellier, cedex 5, France; 3 UMR TETIS, Maison de la Télédétection, 500 rue JF Breton, 34093, Montpellier, cedex 5, France; 4 Medical Microbiology, Radboud University Nijmegen Medical Centre, Nijmegen, The Netherlands

## Abstract

**Background:**

The Camargue region is a former malaria endemic area, where potential *Anopheles *vectors are still abundant. Considering the importation of *Plasmodium *due to the high number of imported malaria cases in France, the aim of this article was to make some predictions regarding the risk of malaria re-emergence in the Camargue.

**Methods:**

Receptivity (vectorial capacity) and infectivity (vector susceptibility) were inferred using an innovative probabilistic approach and considering both *Plasmodium falciparum *and *Plasmodium vivax*. Each parameter of receptivity (human biting rate, anthropophily, length of trophogonic cycle, survival rate, length of sporogonic cycle) and infectivity were estimated based on field survey, bibliographic data and expert knowledge and fitted with probability distributions taking into account the variability and the uncertainty of the estimation. Spatial and temporal variations of the parameters were determined using environmental factors derived from satellite imagery, meteorological data and entomological field data. The entomological risk (receptivity/infectivity) was calculated using 10,000 different randomly selected sets of values extracted from the probability distributions. The result was mapped in the Camargue area. Finally, vulnerability (number of malaria imported cases) was inferred using data collected in regional hospitals.

**Results:**

The entomological risk presented large spatial, temporal and *Plasmodium *species-dependent variations. The sensitivity analysis showed that susceptibility, survival rate and human biting rate were the three most influential parameters for entomological risk. Assessment of vulnerability showed that among the imported cases in the region, only very few were imported in at-risk areas.

**Conclusion:**

The current risk of malaria re-emergence seems negligible due to the very low number of imported *Plasmodium*. This model demonstrated its efficiency for mosquito-borne diseases risk assessment.

## Background

In the past, malaria was endemic and constituted a major health issue in France in marshy areas, particularly the Camargue, which was an active focus until the beginning of the 20^th ^century. Malaria decreased drastically due to the draining of marshes, rearing of livestock, improvement of housing and living conditions and the use of quinine [1]. Malaria disappeared from the Camargue after World War II: the last *Plasmodium vivax *malaria epidemic occurred in 1943, with about 400 estimated cases [2]. Recent entomological surveys reported huge *Anopheles *populations in this area [3-5], and considered *Anopheles (Anopheles) hyrcanus *as being the main potential malarial vector based on its anthropophilic feeding behaviour and abundance [4,6]. Thus, the Camargue is currently facing an "anophelism without malaria" situation. Moreover, autochthonous transmission was recently suspected in the French Mediterranean coast in 2006 [7], supporting the idea that southern France remains suitable for malaria transmission. The number of imported malaria cases have increased dramatically since the 1970s, in parallel with increased international travels, with an average of about 6,400 cases per year for the last ten years in France, leading to a massive *Plasmodium *introduction from endemic countries into France [8,9]. These observations suggest that the malaria situation needs to be re-examined, and the aim of this paper is to infer current risk of malaria re-emergence, to identify hot spots for malaria re-emergence in the Camargue and to develop a generic model for mosquito transmitted diseases.

**Figure 1:**
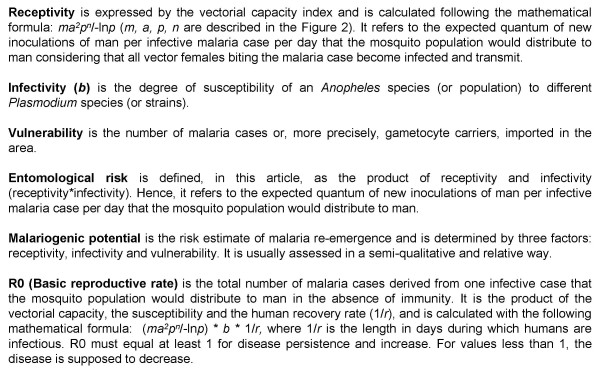
Risk of malaria re-emergence.

**Figure 2:**
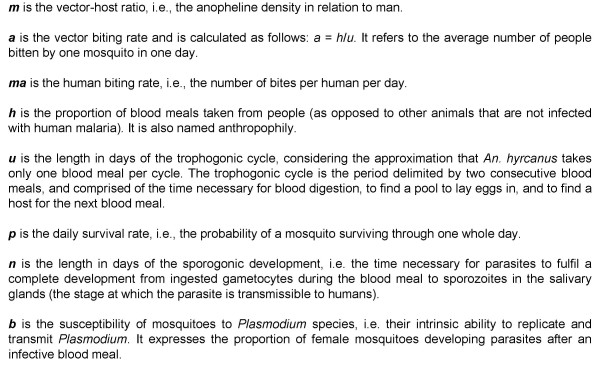
Entomological factors.

The risk of malaria re-emergence in an area (i.e., the recurrence of malaria transmission in an area) may be estimated by three factors: receptivity, infectivity and vulnerability [10-13], usually assessed at the regional scale in a semi-qualitative way (Figure 1, 2) [14]. In this article, a quantitative entomological risk, which is the product of receptivity and infectivity, is calculated and the impact of vulnerability is discussed. The main objective of this work was to estimate the risk of malaria re-emergence at the local scale, considering the temporal and spatial local variations of the three components in order to identify hot spots for malaria resurgence in the Camargue.

The main difficulty that occurs when modeling is the quantification of biological parameters, especially for entomological data, as the field and laboratory studies are very painstaking, time-consuming and only rarely permit conclusions on a precise value. In this article, a probabilistic approach, taking into account the uncertainties and variability of inputs, was applied to a vector-borne disease, which constitutes an innovative method. Even if the malaria situation needs to be re-examined in the Camargue, this disease does not constitute a major health issue. The aim of this article is not to provide a public health tool that can be used to control malaria in the Camargue, but to present an innovative approach to spatialized quantitative risk assessment applied to a vector-borne disease.

Receptivity and infectivity were estimated for the potential vector *An. hyrcanus *as i) it is now considered as the main potential malaria vector and ii) other *Anopheles *species are rare in the Camargue area. However, the approach developed here is applicable to other mosquito species.

## Materials, methods and data processing

### Study area

The Camargue is the main wetland area in Southern France and covers the Rhone river delta. This area has a Mediterranean climate characterized by warm, dry summers and mild, wet winters. Total annual rainfall usually ranges between 500 and 700 mm and occurs mainly in autumn, and the annual mean temperature is 14°C.

Water pools and marshes cover a large part of the Camargue. Water is provided either by rains or a very tight canal network diverted from the river Rhone used to irrigate paddies or to fill marshes. Management of water is at the level of individual field owners depending on use: grazing for horses, cows or sheep, exploitation of reeds or rice, hunting reserves for waterfowl and nature preservation. Landscapes in the Camargue are strongly affected by the duration of submersion and the salinity of the soils. They are organized roughly in a south-north gradient of salinity, with agricultural land and reed marshes in the north and natural salty ponds and salt marshes in the south [15].

Moreover, there are various forms of agriculture (including vineyard, paddies, market gardening, fruit growing and exploitation of reeds) and rice, which covers more than 18,000 hectares in the Camargue, is the main cultivation [16]. Livestock includes horses, cows and sheep.

The Camargue hosts nearly 100,000 permanent inhabitants distributed between towns, hamlets and isolated houses. Moreover, the number of people increases in summer due to tourism.

### Quantitative risk assessment using a probabilistic approach

**Figure 3:**
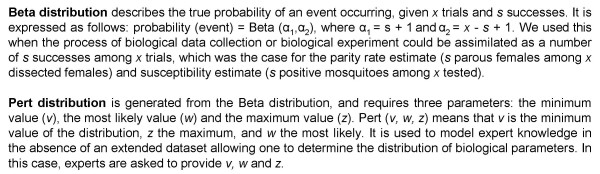
Probability distributions.

The objective of this method was to organize and analyse scientific information in order to infer the risk of malaria re-emergence taking into account the variability and uncertainty of the input components and the final risk estimate. Such analysis, using reiterated simulations, have been performed for a decade for risk assessment in food microbiology, for example [17,18]. Information and data for the development of the entomological risk model were obtained from field surveys, literature, unpublished data and expert opinion. Biological parameters were estimated by probability distributions in a plausible way that is coherent and conceivable and they were fitted with Pert or beta distribution [18,19] (Figure 3). An amount of 10,000 reiterated simulations generated by the Latin Hypercube method associated with the probability distributions was used to describe both variability and uncertainty within the input parameters and the model [20,21]. The outcome is a statistical distribution of risk, as well as a mean value of the risk estimate. Sensitivity analysis was performed to point out factors responsible for the main impact on the risk estimate. The @risk^® ^(Palisade Corporation) software version 4,5,3 was used.

### Meteorological data

Daily temperatures (2005) and mean monthly temperatures (from 2002 to 2006) recorded by MeteoFrance at Aigues-Mortes (western Camargue) and at Tour du Vallat (south-eastern Camargue) were used. Daily temperatures were smoothed with a moving average (running mean) of three days to filter daily variations. For both types of data (daily and monthly), we calculated the average temperature for the Camargue based on the two stations. Humidity was recorded in 2005 at Marais du Vigueirat. These meteorological data were used to estimate some of the biological parameters of *An. hyrcanus*.

### Entomological data: receptivity

In order to assess receptivity, it was necessary to evaluate the human biting rate (ma), the vector biting rate (*a*), the survival rate (*p*) and the sporogonic cycle (*n*) and their spatial and temporal variations (Figure 2).

#### Space and time-dependency

Although we sampled a huge amount of *An. hyrcanus *(125,848 specimens captured, 504 females dissected), it has not been possible to estimate precisely potential spatial variations of some biological parameters. Thus and due to the small size of the Camargue, the biology of *An. hyrcanus *was considered to be homogeneous in the whole area, which means that the vector biting rate (*a*), survival rate (*p*) and sporogonic cycle (*n*) (Figure 2) did not vary spatially. On the contrary, as *An. hyrcanus *presence and density depend on the biotopes and the season [4,5], the vector-host ratio *m *(and hence the human biting rate (*ma*)) (Figure 2) presents a strong spatial heterogeneity (Table 1). Spatial variations were assessed based on a Geographic Information System (GIS) computing data for each 30 meter-wide pixel in the Camargue [22].

**Table 1:** Space, time and *Plasmodium *species-dependent variations in receptivity, infectivity and vulnerability.

		Variation		
		
Components		Space dependent	Time dependent	*Plasmodium*-species dependent
Receptivity	m (vector-host ratio)	YES	YES	NO
	a (vector-biting rate)	NO	YES	NO
	h (anthropophily)	NO	NO	NO
	u (length of the trophogonic cycle)	NO	YES	NO
	p (survival rate)	NO	NO	NO
	n (sporogonic cycle)	NO	YES	YES
Infectivity	b (susceptibility)	NO	NO	YES
Vulnerability		YES	YES	YES

All parameters were considered time-dependent, except the *Anopheles *anthropophily (h) and, as a practical approximation, the survival rate (p) (Table 1). Time-variations, which are detailed below, were described at a monthly time step.

#### Human biting rate (*ma*)

The presence and density of *An. hyrcanus *were inferredusing remote sensing, entomological adults and larvae collections [22]. Analysis of larval data led to the definition of a larval index that was calculated for each pixel in the Camargue based on environmental key factors. An adult abundance index was generated from the larval index and was also calculated for each pixel in the study area. Comparison of the adult abundance index and the maximum number of *An. hyrcanus *captured in the same pixel with CDC-light traps+CO_2 _showed a highly significant linear regression, allowing us to infer, using key environmental factors, the maximum number of *An. hyrcanus *captured during the year with CDC-light traps+CO_2 _for each pixel in the Camargue [22].

The mean annual dynamics of *An. hyrcanus *in the Camargue was inferred from the results of several capture campaigns conducted during several years, in several places, using several capture methods (Table 2). Specimens of this species were collected from March to October, and presented huge abundance variation (none of them were collected during winter). In each capture month, the mean percentage of collected mosquitoes (among the total number of mosquitoes captured during the year) were 0, 0, 0, 4 [1; 7], 12 [2; 21], 66 [51; 80], 18 [9; 27], 0 from March to October, respectively (with the associated confidence interval in brackets), and the maximum number of *An. hyrcanus *captured during the year refers to the month of August. Hence, the spatial distribution of the annual maximum number of *An. hyrcanus *was combined with the mean annual dynamics in order to determine, for each pixel and for each month in the Camargue, the number of *An. hyrcanus *captured with CDC-light traps+CO_2_.

**Table 2:** Total number of *An. hyrcanus *collected per month and associated percentages (bracket)

	Capture places							
	Méjannes	Aimargues	Mourgues	Pont de gau	Carbonnière	Vigueirat	Western Camargue^1^	Tour du Vallat

Year	2005	2005	2005	2005	2005	2005	1969–1994	2004
Capture method	CO2 traps	CO2 traps	CO2 traps	CO2 traps	CDC-LT + CO2	CDC-LT + CO2	Human landing catch	Horse bait trap
Reference	G. L'Ambert, unpublished data	G. L'Ambert, unpublished data	A. Carron, unpublished data	A. Carron, unpublished data	[4]	[4]	EID-Méditérannée, unpublished data^2^	[3]
March	-	-	-	-	0 (0)	5 (0)	(0)	-
April	-	-	-	-	0 (0)	7 (0)	(0)	-
May	1 (0)	0 (0)	2 (1)	2 (0)	16 (0)	296 (0)	(0)	0 (0)
June	27 (0)	0 (0)	18 (7)	28 (3)	673 (12)	6737 (6)	(4)	3 (0)
July	357 (5)	0 (0)	104 (42)	107 (12)	374 (7)	17739 (16)	(11)	38 (1)
August	5933 (83)	102 (95)	94 (38)	730 (84)	3257 (59)	61315 (55)	(43)	4101 (71)
September	857 (12)	5 (5)	32 (13)	7 (1)	1228 (22)	25708 (23)	(42)	1640 (28)
October	0 (0)	0 (0)	0 (0)	0 (0)	3 (0)	124 (0)	(0)	4 (0)
Total	7175	107	250	874	5551	111919		5786

Note:

^1 ^approximatively 12 points

^2 ^mean percentage

From this, the human biting rate (*ma*) was inferred from a comparison between mean CDC-light trap captures and mean human landing captures. Captures conducted the same night, in the same area (Carbonnière vs Marais du Vigueirat) and in pixels of the same adult abundance index class were compared (the following adult abundance thresholds were chosen in order to determine the classes: 10; 100; 500; 1000; 2000; 5000 (Table 3 and 4). For example, human landing captures carried out on the 10^th ^of August in Carbonnière in pixel Hu5 and Hu6 were compared to light trap captures carried out on the same date and in the same area, in pixel LT1, LT4 and LT5 (all of these pixels have an adult abundance index between 100 and 500). Comparison showed a highly significant linear relationship between CDC-light traps and human captures (r^2 ^= 0.66, p < 0.01). Indeed, human biting rate (*ma*) was extrapolated from the following formula: *ma *= (0.39 LT + 0.049)*1.25. As human landing captures refer to the hour following sunset, a correction factor of 1.25 was associated with the estimation of *ma *in order to obtain *ma *for the entire night (Table 3).

**Table 3:** Number of *An. hyrcanus *captured during human landing catches sessions.

Area	Pixel	Adult abundance index	08/08/2005	10/08/2005	19/09/2005	21/09/2005	04/10/2005	23/08/2006	05/09/2006
Vigueirat	Hu1	8676	6312	-	0	-	14	-	-
Vigueirat	Hu2	122	26	-	0	-	0	-	-
Vigueirat	Hu3	308	2	-	9	-	0	-	-
Vigueirat	Hu4	2430	-	-	-	-	-	-	67
Carbonnière	Hu5	242	-	304	-	25	-	-	-
Carbonnière	Hu6	155	-	2	-	1	-	-	-
Carbonnière	Hu6	155	-	0	-	0	-	-	-
Carbonnière	Hu8	687	-	-	-	-	-	39	-
Carbonnière	Hu5	242	-	-	-	-	-	156	-
Carbonnière	Hu7	5348	-	-	-	-	-	16	-
Carbonnière	Hu7	5348	-	-	-	-	-	4	-

Adult abundance index relative to the pixel where the capture was conducted is indicated. Human landing results are relative to the hour following sunset which represent the *An. hyrcanus *aggressiveness peak [4] and approximately 80% of the total number of *An. hyrcanus *captured during the entire night. Each line corresponds to a human landing catch session carried out by one volunteer belonging to the research team (two people were present at Hu6 and Hu7).

**Table 4:** Number of *An. hyrcanus *captured during light trap sessions. Adult abundance index relative to the pixel where the capture was conducted is indicated.

Area	Pixel	Adult abundance index	08/08/2005	10/08/2005	19/09/2005	04/10/2005	23/08/2006	05/09/2006	21/09/2005
Vigueirat	LT1	346	218	-	346	6	-	-	-
Vigueirat	LT2	208	70	-	208	1	-	-	-
Vigueirat	LT3	6594	3546	-	6594	20	-	-	-
Vigueirat	LT4	10920	15971	-	10920	15	-	-	-
Vigueirat	LT5	4898	2016	-	4898	32	-	-	-
Vigueirat	LT6	1769	180	-	1769	3	-	1200	-
Vigueirat	LT7	3732	3645	-	3732	4	-	-	-
Vigueirat	LT8	2681	1006	-	2681	24	-	-	-
Carbonnière	LT1	492	-	254	-	-	-	-	8
Carbonnière	LT2	1299	-	102	-	-	101	-	10
Carbonnière	LT3	1250	-	39	-	-	-	-	0
Carbonnière	LT4	433	-	20	-	-	-	-	3
Carbonnière	LT5	176	-	288	-	-	-	-	3
Carbonnière	LT6	705	-	30	-	-	78	-	19
Carbonnière	LT7	721	-	17	-	-	-	-	15
Carbonnière	LT8	0	-	1	-	-	-	-	0
Carbonnière	LT9	5348	-	-	-	-	28	-	-

Light trap captures are relative to the entire night.

**Figure 4:**
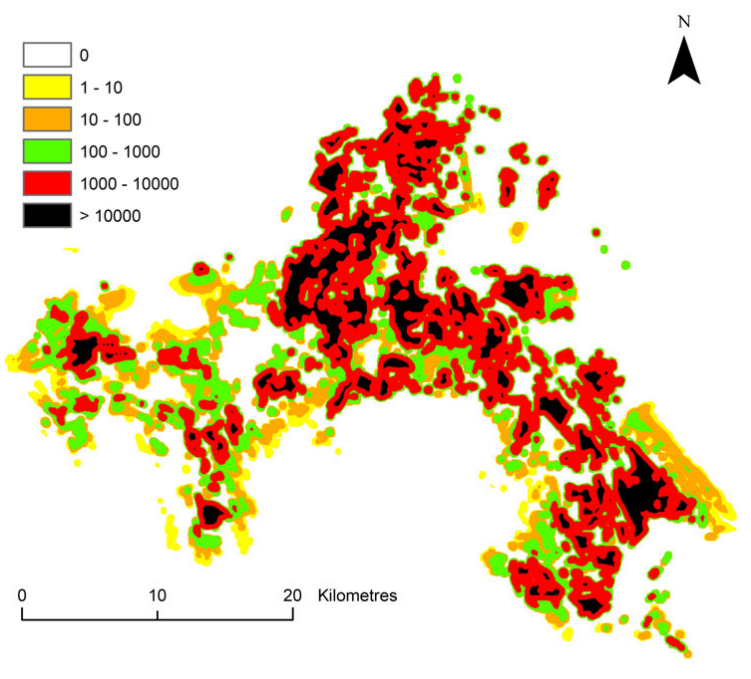
**Spatial distribution of the human biting rate in the Camargue in August**. Classes were arbitrary chosen with a logarithmic scale.

The result is the spatio-temporal distribution of *ma *in the Camargue, i.e., an estimation of the human biting rate for each 30 m × 30 m pixel and each month. The human biting rate was figured for the month of August in order to illustrate this paragraph (Figure 4). Variability and uncertainty were taken into account for each step leading to the assessment of variability and uncertainty of *ma *for each pixel.

#### *Anopheles hyrcanus *anthropophily (*h*)

**Figure 5:**
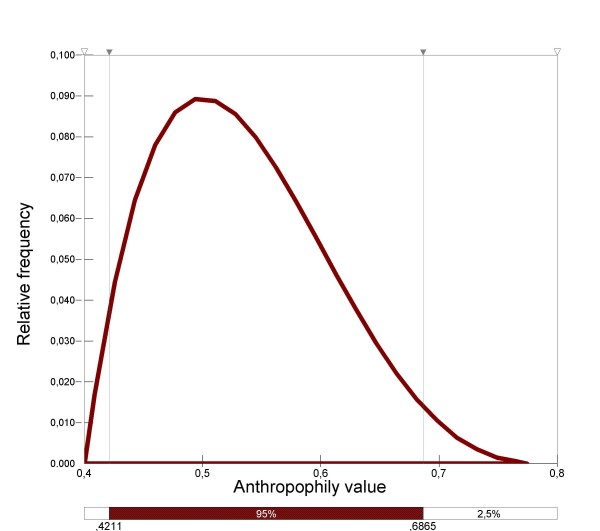
Distribution of *An. hyrcanus *anthropophily.

(Figure 2) was estimated from the comparison between human landing, light traps and horse bait trap results for *An. hyrcanus *and other *Anopheles *species [4], and was fitted as follows: Pert distribution (0.4; 0.5; 0.8)(Figure 5). It was assumed that *h *did not vary throughout the year.

#### The length of the trophogonic cycle (*u*)

(Figure 2) was calculated using the following formula, which estimates the length of time of blood digestion: *u *= *f*_1_/(*T-g*_1_) where *f*_1 _and *g*_1 _are factors depending on humidity and *T *is the temperature. *f*_1 _and *g*_1 _were experimentally determined and evaluated at 36.5°C-days and 9.9°C, respectively, when the humidity reached 70–80% (from June to September 2005, mean monthly humidity varied from 63 to 90% in the Camargue) [23]. Moreover, Detinova assumed that it was reliable to add 24 hours to take into account the time necessary to find a host and the time necessary to find a pool to lay eggs in, in order to fulfil a complete gonotrophic cycle [23]. Considering expert knowledge and bibliographical data [24], a two day range of values was fixed for each month in the Camargue, and the length of the trophogonic cycle was fitted as indicated in Table 5.

**Table 5:** Length of the trophogonic cycle

Month	Mean temperature (2002–2006)	Length of trophogonic cycle (from Shlenova and Detinova)	Pert distribution
March	10.9	38.4	
April	14	9.9	(9; 10; 11)
May	17.8	5.6	(4.5; 5.5; 6.5)
June	23	3.8	(3; 4; 5)
July	24.8	3.4	(3; 3.5; 5)
August	24	3.6	(3; 3.5; 5)
September	20.3	4.5	(3.5; 4.5; 5.5)
October	17.1	6.1	(5; 6; 7)

**Figure 6:**
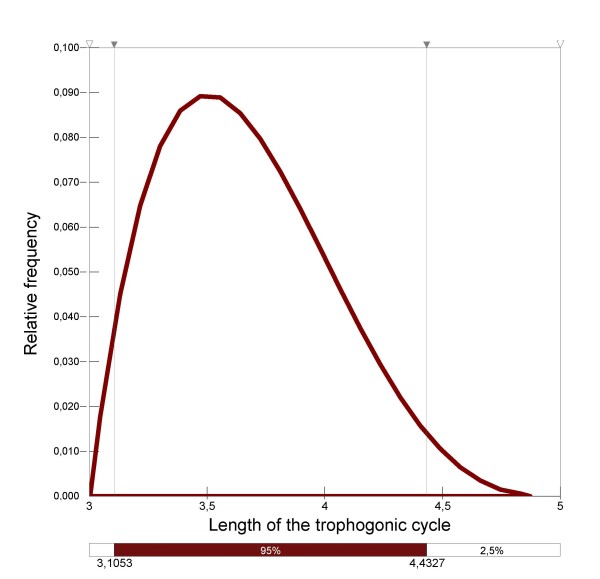
Distribution of the length, in days, of the trophogonic cycle in August.

In order to illustrate this, the distribution of the length of the trophogonic cycle was determined for August (Figure 6).

#### The daily survival rate (*p*)

(Figure 2) was estimated from parity rates (P) using the following formula: *p *= P^1/u ^[25] which is relevant in the case of stable populations. Considering that the *An. hyrcanus *population increases and decreases progressively according to the *Anopheles *biology, the approximation that populations were stable during the summer was used [5]. Parity rates observed in June, July and September 2005 in Carbonnière and Marais du Vigueirat were used (due to the low number of dissected mosquitoes, the parity rate calculated in June in Carbonnière was not included) [4]. They were fitted as indicated in Table 6, and the mean value was calculated from the five probability distributions in order to obtain the mean *An. hyrcanus *parity rate in the Camargue from June to September 2005.

**Table 6:** Parity rates observed in the Camargue in 2005

	Carbonnière	Marais du Vigueirat
June	-	Beta (18; 9)
July	Beta (20; 69)	Beta (29; 91)
September	Beta (89; 45)	Beta (40; 80)

**Figure 7:**
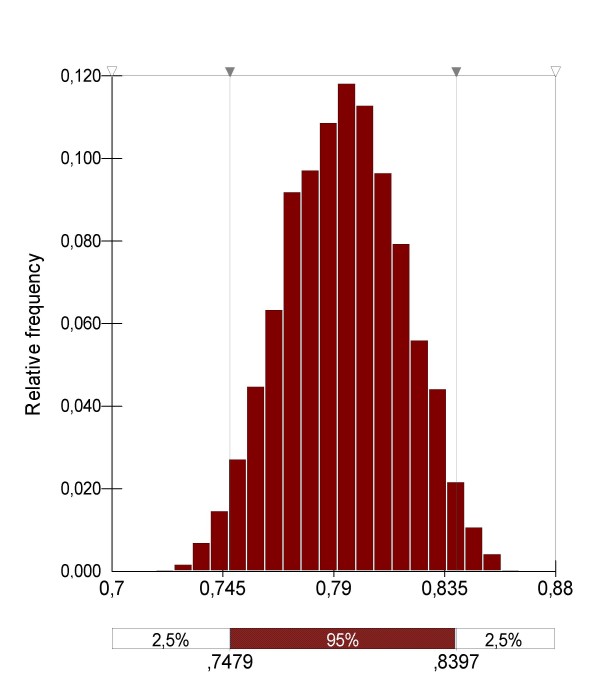
Distribution of the *An. hyrcanus *daily survival rate.

During the same period (June to September 2005), the daily temperature varied from 18.5°C to 26.4°C, with a mean value of 23.2°C. The length of the trophogonic cycle, calculated using the same method as before, ranged from 3.2 days to 5.2 days, with a mean value of 3.7, and was fitted as follows: Pert distribution (3; 3.5; 5.5). Using the formula *p *= P^1/u^, a mean *p *value of 0.79 was obtained (Figure 7). Due to difficulty in obtaining a precise value for parity rates for the entire year, it was considered that *p *did not vary from March to October in this model.

#### The length of the sporogonic period (*n*)

(Figure 2) was calculated as follows: *n *= *f*_2_/(*T-g*_2_), where *T *is temperature and *f*_2 _and *g*_2 _are *Plasmodium *species-dependent factors [26]. *f*_2 _and *g*_2 _were experimentally evaluated at 111°C-days and 16°C, 105°C-days and 14.5°C for *Plasmodium falciparum *and *P. vivax*, respectively. However, Grassi and MacDonald estimated threshold temperatures under which the sporogonic development is not completed: 18–19°C for *P. falciparum*, 15–17°C for *P. vivax *[24,27].

### Entomological data: infectivity

**Figure 8:**
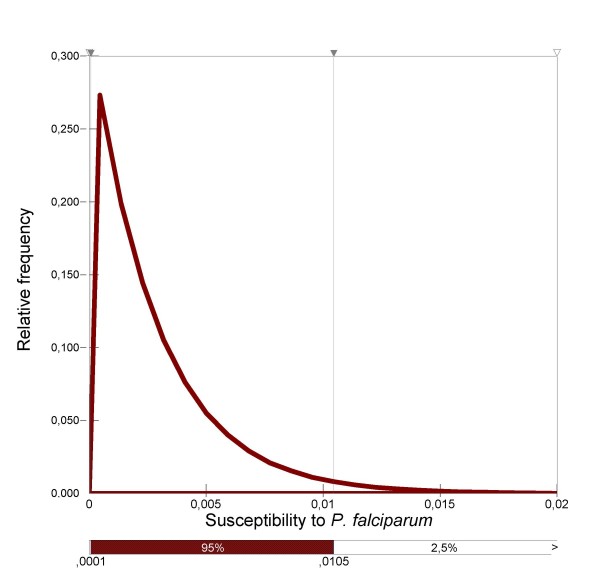
Distribution of *An. hyrcanus *susceptibility to *P. falciparum*.

*Anopheles hyrcanus *susceptibility was estimated based on data derived from experimental membrane-feeding experiments conducted with cultured *P. falciparum *in the laboratories of Radboud University Nijmegen Medical Centre (The Netherlands) following a routine protocol [28,29]. Among 350 *An. hyrcanus *tested, none were found able to transmit tropical *P. falciparum *strains. Hence, *An. hyrcanus *susceptibility to *P. falciparum *was fitted with a beta distribution: Beta (1; 351) (Figure 8).

**Figure 9:**
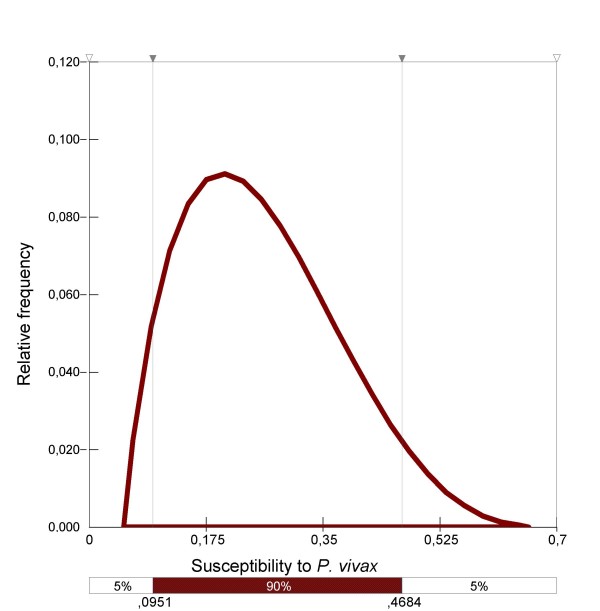
Distribution of *An. hyrcanus *susceptibility to *P. vivax*.

*Anopheles hyrcanus *susceptibility to *P. vivax *has not yet been tested, but this species has been considered a vector of *P. vivax *in Afghanistan for more than 30 years [30,31]. Genetic comparison based on ITS2 sequences between French, Afghan, Turkish and Iranian specimens concluded that they were identical. Hence, it was considered that *An. hyrcanus *was susceptible to tropical *P. vivax *strains, and estimation of its susceptibility, based on expert knowledge, was fitted as follows: Pert distribution (0.05; 0.20; 0.70) (Figure 9).

*An. hyrcanus *susceptibility to either *P. falciparum *or *P. vivax *was considered to be homogeneous through time and space (Table 1).

*Anopheles hyrcanus *susceptibility to *Plasmodium ovale *and *Plasmodium malariae *was not inferred, as very little is known about European *Anopheles *susceptibility to these two species.

### Parasitological data: vulnerability

Vulnerability is related to gametocyte carriers as the gametocyte is the stage transmissible to mosquitoes. Nevertheless, very little is known about gametocyte carriers, and any information must be considered in the context of imported malaria cases, i.e. taking account of the fact that the illness is diagnosed and treated. Since anti malarial-treatment in France is generally conducted with drugs that do not prevent gametocyte emergence [8], an assumption that every patient may develop some gametocytes was made. Hence, we used imported malaria cases to approach the gametocyte presence in the Camargue.

Data relative to imported malaria cases were obtained from five public hospitals localized in important towns in/around the Camargue (Montpellier, Nîmes, Avignon, Arles and Marseille). These data were analysed with regard to the date, *Plasmodium *species, patients' residence for the 2004–2005 period and contamination place.

It was estimated that imported malaria case data obtained from public hospitals represented about 50–55% of all imported malaria cases, with other cases being diagnosed by private laboratories [32,33]. Thus, the total number of imported malaria cases was estimated using a 52.5% correcting factor assuming that epidemiological data provided by public hospitals were representative of the total number of imported malaria cases.

## Results

### Vulnerability

In 2004 and 2005, 657 imported cases were diagnosed in the region (corresponding to a total of 1251 estimated imported cases), among which *P. falciparum, P. vivax, P. ovale *and *P. malariae *represented 85.5%, 7%, 5.5% and 2%, respectively. Moreover, 35% of these cases occurred between August and September. Among 528 patients for whom the place of residence was known, 75% were living in Montpellier, Nîmes, or Marseille big cities, and only 7 patients were living in areas where *An. hyrcanus *could be present.

Countries in which patients were infected were not known for 2004–2005. For the 2001–2003 period, 96% of imported malaria cases were contracted in Africa, and the Comoros Islands represented the main place of contamination for cases diagnosed in Marseille, Arles and Nîmes. Data collected in Montpellier and Avignon showed that imported cases were mainly contracted in West Africa.

### Entomological risk (receptivity*infectivity)

**Figure 10:**
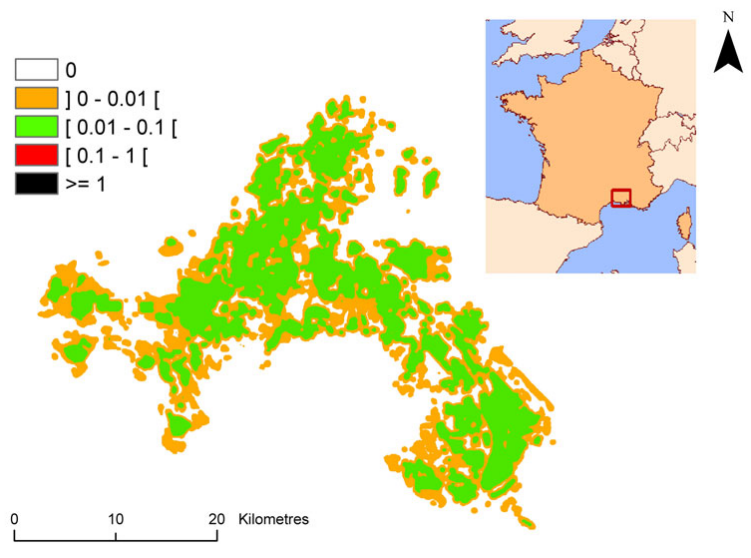
**Spatial variations of *P. falciparum *transmission risk estimate (ranging from 0 to more than 1) in June in the Camargue**. Classes were arbitrary chosen with a logarithmic scale.

**Figure 11:**
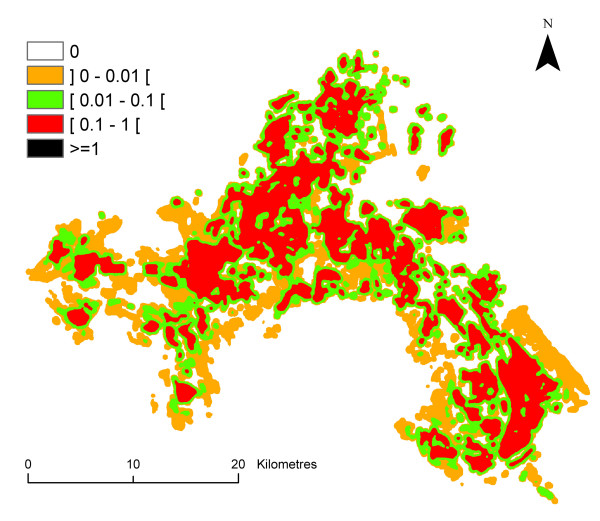
**Spatial variations of *P. falciparum *transmission risk estimate (ranging from 0 to more than 1) in July in the Camargue**. Classes were arbitrary chosen with a logarithmic scale.

**Figure 12:**
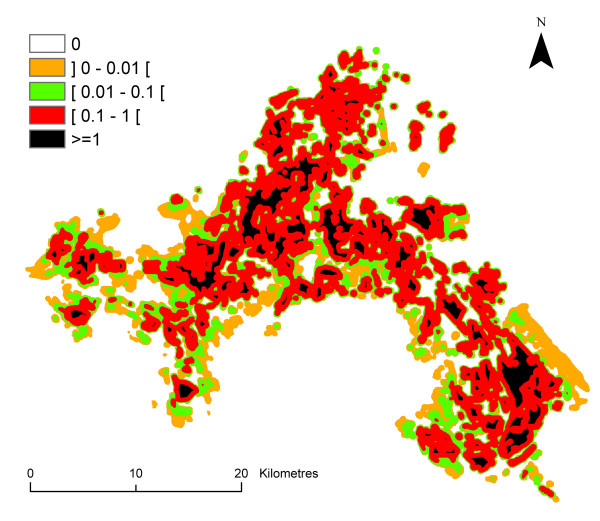
**Spatial variations of *P. falciparum *transmission risk estimate (ranging from 0 to more than 1) in August in the Camargue**. Classes were arbitrary chosen with a logarithmic scale.

**Figure 13:**
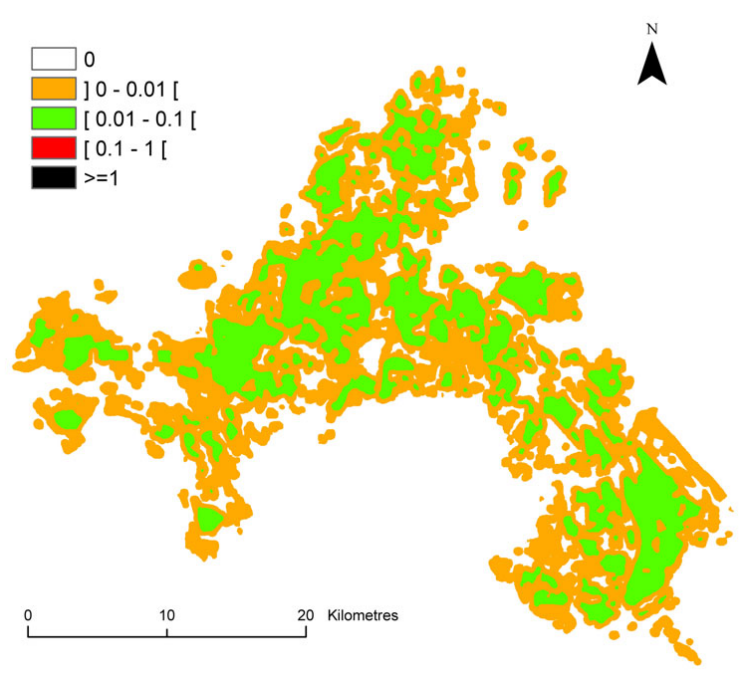
**Spatial variations of *P. falciparum *transmission risk estimate (ranging from 0 to more than 1) in September in the Camargue**. Classes were arbitrary chosen with a logarithmic scale.

**Figure 14:**
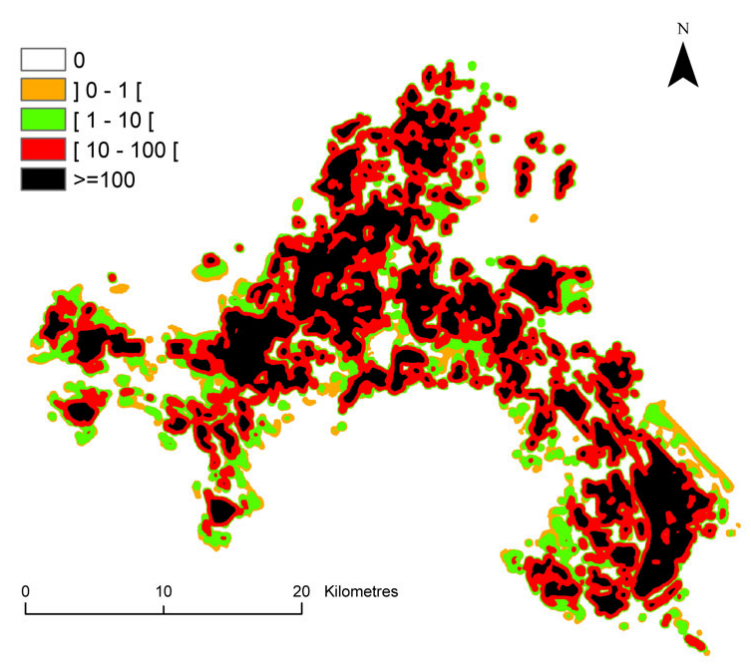
**Spatial variations of the *P. vivax *transmission risk estimate (ranging from 0 to more than 100) in August in the Camargue**. Classes were arbitrary chosen with a logarithmic scale.

The mean value of the entomological risk was assessed for *P. falciparum *species from June to September, and for *P. vivax *for the month of August for each pixel of the Camargue map (Figure 10, 11, 12, 13, 14) (the entomological risk was not assessed for *P. ovale *and *P. malariae *due to the low number of imported cases and the lack of information concerning both species). It was calculated with 10,000 different randomly selected sets of values extracted from input distributions.

Strong differences were observed in the entomological risk for the two *Plasmodium *species: *P. falciparum *transmission risk estimate ranges from 0 to more than 1 although *P. vivax *transmission risk estimate ranges from 0 to more than 100.

### Uncertainties of the risk estimate and sensitivity analysis

For each pixel the outcome of the model is a statistical distribution of the risk estimate, generated by variability and uncertainty within inputs. For example, the entomological risk estimate ranges from about 9.10^-6 ^to 20, with 95% of the values being between 0.016 and 4.7 for pixel having a mean value of about 1.

**Figure 15:**
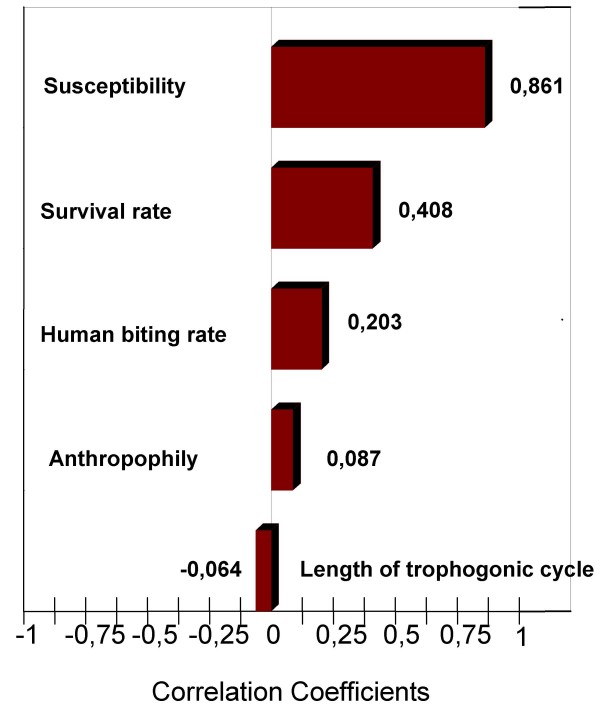
Sensitivity analysis.

The sensitivity analysis was conducted for pixels of which the mean entomological risk estimate is about 1. Results of the sensitivity analysis show the correlation coefficients between different varying inputs and the consecutive varying risk estimate (Figure 15).

Sensitivity analysis carried out for pixels presenting a lower risk estimate showed a predominance of susceptibility. However, correlation coefficients of the survival rate and the human biting rate were approximately equal. Sensitivity analysis carried out for the *P. vivax *entomological risk estimate showed the equal importance of the susceptibility, the survival rate and the human biting rate.

## Discussion

The entomological risk index used in this article refers to the risk of transmission (Figure 1): the risk of transmission being high when the entomological risk is high. Results clearly indicate a maximum risk of *P. falciparum *transmission in August in the Camargue considering the spatial distribution and value of the entomological risk estimate, which results from length of the trophogonic cycle and sporogonic period and the human biting rate. The length of the trophogonic cycle and sporogonic development period are directly influenced by the mean temperature in our model, and the human biting rate depends directly on the *An. hyrcanus *dynamics and density [5]. The human biting rate being the only space-dependant factor in our model, one could be tempted to approach roughly the risk of transmission by the human biting rate. Nevertheless, the entomological risk presents also temporal variations, which depend not only on the human biting rate but also on the impact of temperature on the length of the trophogonic and sporogonic cycle. Dynamics of *An. hyrcanus *and mean temperature do not evolve exactly in the same way in the Camargue, which shows the necessity of estimating all the parameters of the entomological risk.

The risk of *P. vivax *transmission is more than one hundred times higher than the risk of *P. falciparum *transmission, which is due to infectivity and the length of sporogonic cycle differences.

Nevertheless, the entomological risk calculated herein is a theoretical index as the human biting rate reaches more than 10,000 bites per human per night reflecting the abundance of *An. hyrcanus*. This will, of course, never happen as no one can endure such a large number of bites. This suggests that, in the future, it could be necessary to combine the entomological risk with human presence and exposure to mosquito bites in order to evaluate the real human biting rate. Such analyses would require complementary geographical and sociological studies.

Although all of France faces a large number of imported cases [8], particularly in the south-east of the country, vulnerability in at risk areas is very low because most imported cases are present in large cities.

The entomological risk, referring to the risk of transmission in this article (Figure 1), has to be interpreted in a relative way due to its definition and the estimation of its parameters. Thus, this study underlined space, time and *Plasmodium *species-dependant of the risk of potential transmission. Of course, the risk of potential transmission is connected with the risk of malaria re-emergence when gametocytes carriers are introduced within at risk areas. Considering the entomological risk and the length in days of the infectious period of humans would allow estimation of R0 and the following absolute risk of malaria re-emergence. Finally, the current risk of malaria re-emergence seems negligible due to the very low number of imported *Plasmodium*.

As stated in the introduction, the aim of this study was not to build a public health tool for controlling malaria in the Camargue. The emphasis was on presenting an innovative approach of spatialized quantitative risk assessment applied to a vector-borne disease, which has not been previously conducted. The main advantages of such a probabilistic approach are the possibility of integrating the uncertainty and variability of inputs within a model and to quantify the uncertainty of the final risk estimate. The deterministic approaches used thus far have not taken uncertainty and variability into account [27,34-36] and have produced precise outcomes, which could lead to misinterpretation as the final risk estimate could vary significantly due to input variability. Integration of uncertainty and variability in deterministic models would rapidly lead to complicated models, requiring laborious mathematical developments [18].

Moreover, the approach applied in this study is based on distributions that are combined, resulting in quite sophisticated analyses that are intuitive and easily understood. The uncertainty within the risk estimate is a crucial point for decision makers, which usually apply some rough "safety margin" around a deterministic estimate to express their feeling of uncertainty. The pros of the approach developped in this article are rightly to quantify this uncertainty.

Such a method could be applied to other areas where malaria is still a threat or to emerging vector-borne diseases, such as the dengue or chikungunya virus infections. This method could be used in a controlled way, in order to identify areas and time periods that correspond to the highest risk of transmission, and to focus control measures where and when transmission is elevated.

It has been shown recently the impact of anthropogenic changes on potential malaria vectors in the Camargue over the last 60 years [37]. This method could be used also to predict the probable impact of future decisions concerning land use for example, and could be a useful tool for decision makers.

The sensitivity analysis underlined factors responsible for entomological risk uncertainty and variation, of which susceptibility, the survival rate and the human biting rate of *An. hyrcanus *have a major impact in this model. Variability in either of these parameters leads to variability and uncertainty in the risk estimate. In contrast to the susceptibility, survival rate and human biting rate, variability in the anthropophily range and the length of the trophogonic cycle range has only a minor impact on the risk estimate. The sensitivity analysis thus appears to be a tool particularly useful for the identification of key factors, which need to be assessed in field surveys.

In the context of emerging vector-borne diseases, emphasis is currently on developing and improving such quantitative risk assessment models integrating variability and uncertainty of biological parameters, which are usually difficult to assess, and especially parameters recorded in the field.

## Competing interests

The authors declare that they have no competing interests.

## Authors' contributions

NP designed the study, analysed the data and drafted the manuscript. AT analysed the data and carried out spatial analysis. CT participated in data collection and analysis. AL managed the susceptibility trials. DF conceived of the study, participated in its design and coordination and helped to draft the manuscript. All authors read and approved of the final manuscript.
